# Attenuated viral hepatitis in *Trem1*−/− mice is associated with reduced inflammatory activity of neutrophils

**DOI:** 10.1038/srep28556

**Published:** 2016-06-22

**Authors:** Jan-Hendrik Kozik, Tanja Trautmann, Antonella Carambia, Max Preti, Marc Lütgehetmann, Till Krech, Christiane Wiegard, Joerg Heeren, Johannes Herkel

**Affiliations:** 1 University Medical Centre Hamburg-Eppendorf, Department of Medicine I, 20246 Hamburg, Germany; 2 University Medical Centre Hamburg-Eppendorf, Institute of Medical Microbiology, Virology and Hygiene, 20246 Hamburg, Germany; 3 University Medical Centre Hamburg-Eppendorf, Institute of Pathology, 20246 Hamburg, Germany; 4 University Medical Centre Hamburg-Eppendorf, Institute of Biochemistry and Molecular Cell Biology, 20246 Hamburg, Germany

## Abstract

TREM1 (Triggering Receptor Expressed on Myeloid Cells 1) is a pro-inflammatory receptor expressed by phagocytes, which can also be released as a soluble molecule (sTREM1). The roles of TREM1 and sTREM1 in liver infection and inflammation are not clear. Here we show that patients with hepatitis B virus (HBV) or hepatitis C virus (HCV) infection manifest elevated serum levels of sTREM1. In mice, experimental viral hepatitis induced by infection with Lymphocytic Choriomeningitis Virus (LCMV)-WE was likewise associated with increased sTREM1 in serum and urine, and with increased TREM1 and its associated adapter molecule DAP12 in the liver. *Trem1*−/− mice showed accelerated clearance of LCMV-WE and manifested attenuated liver inflammation and injury. TREM1 expression in the liver of wild-type mice was mostly confined to infiltrating neutrophils, which responded to LCMV by secretion of CCL2 and TNF-α, and release of sTREM1. Accordingly, the production of CCL2 and TNF-α was decreased in the livers of LCMV-infected *Trem1*−/− mice, as compared to LCMV-infected wildtype mice. These findings indicate that TREM1 plays a role in viral hepatitis, in which it seems to aggravate the immunopathology associated with viral clearance, mainly by increasing the inflammatory activity of neutrophils.

Cells of the innate immune system express extra- and intracellular receptors, such as Toll-like receptors (TLRs) and nucleotide-binding oligomerization domain (NOD)-like receptors, that can sense molecules, which signify the presence of pathogens or occurrence of host cell damage^1^. In recent years, another class of conserved receptors has been identified, the so called triggering receptors expressed on myeloid cells (TREM), which seem to have the capacity to integrate and modulate the signals induced by TLRs and NOD-like receptors^2^,^3^. However, the expression of TREMs seems to be restricted to cells of the myeloid lineage^4^, unlike the widespread expression of TLRs and NOD-like receptors. Several TREM receptors and TREM-like transcripts have been described in humans and mice, which are encoded by clustered genes^5^,^6^.

TREM1 is the best characterized member of the TREM family of receptors that seems to have an activating function^2^,^4^. TREM1 consists of an extracellular Ig V-type domain, a transmembrane region and a short intracellular tail that can recruit the DAP12 adapter molecule for signalling^3^,^6^. TREM1 is constitutively expressed by neutrophils and a subset of monocytes^4^,^7^; its expression can be further up-regulated by microbial components^8^. Studies on TREM1 function have mostly relied on the use of TREM1/Ig fusion proteins or synthetic peptide mimics of TREM1 to block TREM1 signalling, or anti-TREM1 antibodies to activate TREM1^2^,^4^. Ligation of TREM1 has been shown to greatly amplify the secretion of proinflammatory cytokines in response to microbial products^8^. However, the precise role of TREM1 in inflammation and inflammatory diseases has been difficult to characterize, because a physiological ligand for TREM1 has not yet been clearly defined, although several putative ligands have been suggested^9^,^10^,^11^,^12^. Moreover, a recent publication suggested that anti-TREM1 antibodies may have agonistic or antagonistic activity, depending on the assay conditions^12^, thus making it difficult to interpret the findings obtained with such antibodies. The biology of TREM1 is further complicated by the fact that TREM1 can be shed from the cell membrane as a soluble form of TREM1 (sTREM1)^13^, which can serve as a diagnostic marker for infection^14^. The biological function of sTREM1 is thus far not clear^2^,^15^; however, it is conceivable that sTREM1 may act as a negative regulator of TREM1 signalling by neutralising TREM1 ligands. Recently, TREM1-deficient (*Trem1*−/−) mice have been generated that, surprisingly, did not manifest impaired control of infections^7^. Instead, *Trem1*−/− mice exhibited reduced immunopathology and attenuated disease severity^7^.

Although TREM1 activity was initially described in the context of bacterial or fungal infection^2^, a possible role of TREM1 also in viral infections is emerging^15^. It has been observed that at least some *filoviridae* can induce TREM1 signalling and shedding of sTREM1 by human neutrophils^16^. Dengue virus infection was also found to be associated with elevated plasma levels of sTREM1^17^. Thus far, however, it is unclear whether TREM1 activation is a common event in viral infections and whether TREM1 activity can modulate virus-associated inflammation^15^.

It has been reported that several cell types in the liver can express TREM1, including Kupffer cells^11^, sinusoidal endothelial cells^18^ and hepatic stellate cells^19^. Thus, it is conceivable that viral hepatitis might well be suited for clarifying the putative role of TREM1 in viral infections. Viral infections of the liver are a major cause of illness and death worldwide. In particular, virus-induced hepatitis, leading to chronic disease in hundreds of millions of people, is one of the most common causes of liver cirrhosis and liver cancer^20^. After infection with hepatitis viruses, some individuals are able to clear the infection, whereas others remain infected and manifest chronic liver inflammation^21^. CD8+ T cells are the major effector cells that mediate viral clearance from the liver by removal of infected cells; how the innate immune system impacts on viral hepatitis is less clear^21^. Infection of mice with Lymphocytic Choriomeningitis Virus (LCMV) of the strain WE can serve as a mouse model of acute viral hepatitis^22^,^23^,^24^. LCMV, like the common human hepatitis viruses, causes a non-cytopathic infection, in which the induced liver damage is mediated almost entirely by the antiviral immune response^22^, notably by CD8+ T cells, which are essential for the elimination of the virus^25^.

To investigate the role of TREM1 in viral hepatitis, we assessed 1) the plasma levels of sTREM1 in human hepatitis virus infection and in murine LCMV infection, and 2) the effect of TREM1 deficiency on murine LCMV hepatitis in *Trem1*−/− mice. Here we show that sTREM1 is elevated in viral hepatitis, both in humans and in mice. TREM1 deficient mice exhibited accelerated viral clearance, but also reduced immunopathology in the liver. During acute LCMV hepatitis in wild-type mice, TREM1 expression in the liver was mainly confined to neutrophils, which responded to LCMV stimulation by secreting inflammatory cytokines. Accordingly, *Trem1*−/− mice exhibited lower cytokine levels in LCMV-infected livers than wild-type mice. These findings demonstrate a role of TREM1 in viral hepatitis and indicate that interference with TREM1 may attenuate immunopathology associated with viral hepatitis.

## Results

### Human viral hepatitis and murine LCMV hepatitis are characterized by increased sTREM1 levels

**Figure 1:**
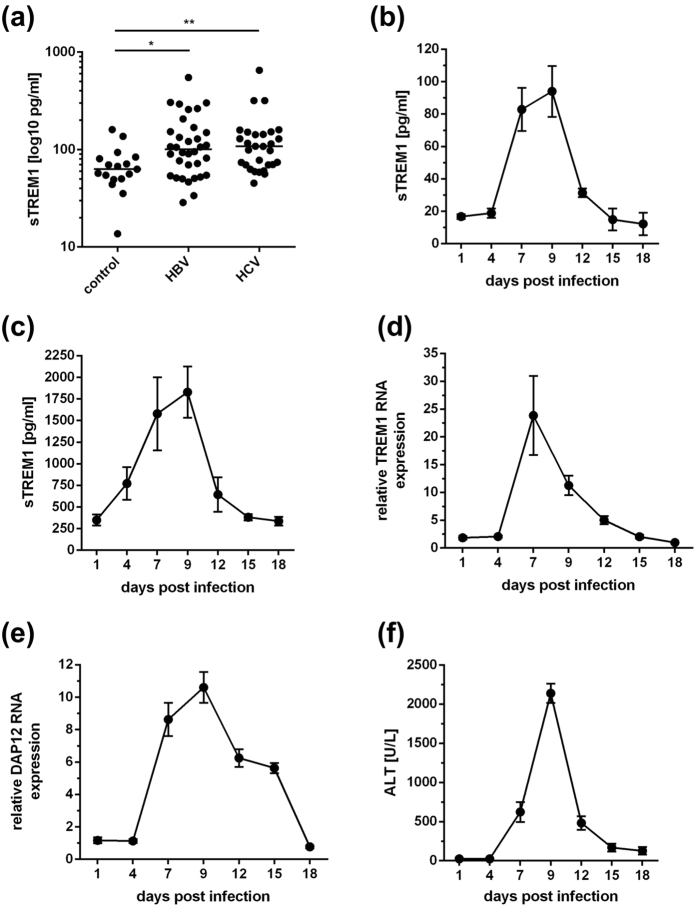
Viral hepatitis is associated with increased TREM1 expression and sTREM1 release. (**A**) Increased sTREM1 concentrations in blood plasma from patients with hepatitis B (HBV; n = 34) or hepatitis C virus infection (HCV; n = 29), as compared to healthy control subjects (n = 17). (**B**) Increased sTREM1 concentrations in blood plasma from C57BL/6 mice during acute hepatitis caused by LCMV-WE infection. (**C**) Increased sTREM1 concentrations in urine from C57BL/6 mice during acute hepatitis caused by LCMV-WE infection. (**D**) Increased expression of TREM1 RNA in infected livers of C57BL/6 mice during acute hepatitis caused by LCMV-WE infection. (**E**) Increased expression of DAP12 RNA in infected livers of C57BL/6 mice during acute hepatitis caused by LCMV-WE infection. (**F**) Increased alanine aminotransferase (ALT) concentrations in blood plasma of C57BL/6 mice during acute hepatitis caused by LCMV-WE infection. *p < 0.05, **p < 0.01.

To assess the possible role of TREM1 in viral hepatitis, we first determined the plasma levels of sTREM1 in patients infected with the hepatitis B virus (HBV; n = 34) or with the hepatitis C virus (HCV; n = 29) and healthy subjects (n = 17); the patient characteristics are depicted in Table 1. Both, HBV- and HCV-infected patients manifested significantly elevated sTREM1 levels, as compared to healthy subjects (Fig. 1A; p < 0.05), indicating that viral hepatitis is associated with shedding of sTREM1. To confirm these findings and correlate sTREM1 levels with the course of infection, we also assessed sTREM1 levels in plasma (Fig. 1B) and urine (Fig. 1C) during acute LCMV hepatitis in C57BL/6 mice over eighteen days after LCMV inoculation, during which LCMV is cleared^24^. The elevation of sTREM1 seemed to follow the elevated expression of TREM1 in the livers of LCMV-infected mice (Fig. 1D). Moreover, sTREM1 elevation seemed to correlate with elevated expression of DAP12 in the livers of LCMV-infected mice (Fig. 1E), as well as with elevated plasma levels of ALT (Fig. 1F), a marker of hepatocellular damage. These findings indicated that viral hepatitis is characterised by up-regulated TREM1 and DAP12 expression in inflamed livers and shedding of sTREM1.

### *Trem1*−/− mice manifest attenuated LCMV hepatitis and accelerated viral clearance

**Figure 2:**
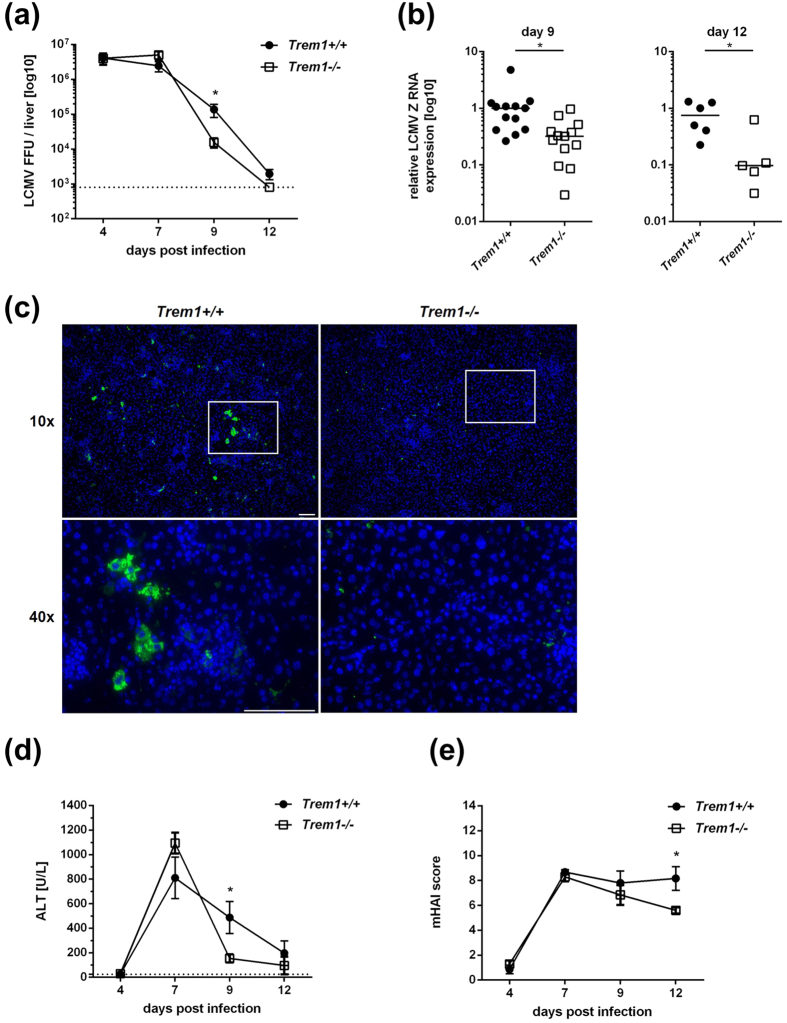
Accelerated viral clearance and reduced liver damage in LCMV-infected *Trem1*−/− mice. (**A**) At the indicated time-points after LCMV inoculation, livers of *Trem1*+/+ or *Trem1*−/− mice were homogenized and the virus titer was determined by the Focus Forming Assay. (**B**) Reduced viral load of *Trem1*−/− livers at day 9 and 12 after LCMV inoculation was confirmed by quantitative RT-PCR analysis for the LCMV Z RNA. (**C**) Frozen liver sections taken at day 9 after LCMV inoculation were stained for the LCMV nucleoprotein with VL4 antibody (green); nuclei were stained with Hoechst 33258 (blue). (**D**) Protracted ALT elevation in blood plasma of *Trem1*+/+ mice at day 9 after LCMV inoculation, as compared to *Trem1*−/− mice. (**E**) Increased histological activity of *Trem1*+/+ mice at day 12 after LCMV inoculation, as compared to *Trem1*−/− mice, determined as the modified histological activity index (mHAI) score. *p < 0.05, **p < 0.01.

To investigate the functional role of TREM1 in viral hepatitis, we infected wild type C57BL/6 mice or *Trem1*−/− mice with LCMV-WE at a dose of 10^6^ FFU. At various days after infection (days 4, 7, 9 and 12), we determined the virus titre in the livers with the focus-forming assay (Fig. 2A). At days 4 and 7, there was no difference in the infection rate in the livers from wild-type or *Trem1*−/− mice. However, at day 9, *Trem1*−/− mice manifested a significantly lower degree of infection, compared to wild-type mice (p < 0.05); at day 12, *Trem1*−/− mice had already cleared the virus, whereas wild-type mice still showed a low level of infection. To confirm these findings, we performed a quantitative RT-PCR of liver RNA for the LCMV Z protein at days 9 and 12 after LCMV inoculation (Fig. 2B). At both time-points, expression of Z protein was significantly increased in the livers of wild-type mice (p < 0.05). We further confirmed these findings by histological staining of liver sections for the LCMV nucleoprotein with VL4 antibody (Fig. 2C). At day 9 after infection, C57BL/6 mice still manifested VL4 positive hepatocytes; in contrast, the number of VL4 positive hepatocytes in *Trem1*−/− mice was negligible. Thus, TREM1 deficiency seemed to induce accelerated clearance of LCMV from the liver.

As it has been reported that TREM1 deficiency can attenuate disease severity in several types of infection^7^, we also determined the plasma ALT levels, as a marker of hepatocellular damage (Fig. 2D), and a pathologist who was blinded to the experimental groups scored the modified histological activity index (mHAI; Fig. 2E). Consistent with the protracted viral hepatitis shown above, wild-type mice displayed significantly protracted ALT elevation and histological activity. Thus, TREM1 deficiency seemed to attenuate LCMV-induced hepatitis.

### TREM1 deficiency does not influence the anti-viral CD8 T cell response

**Figure 3:**
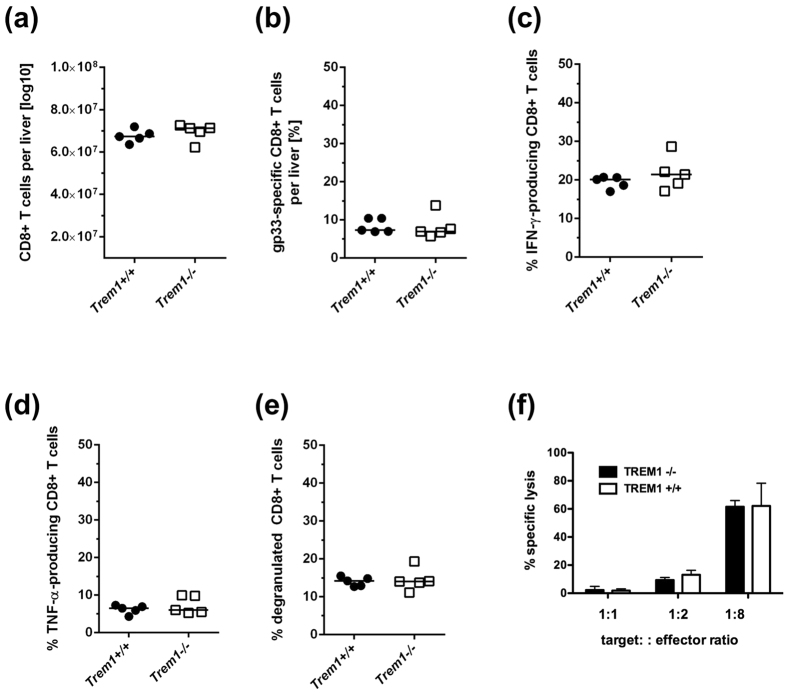
TREM1 deficiency does not influence the anti-viral CD8 T cell response. (**A**) Equal numbers of infiltrating CD8 T cells in LCMV-infected livers of *Trem1*+/+ or *Trem1*−/− mice at day 9 of infection. Each dot represents the absolute number of liver-infiltrating mononuclear cells of one individual mouse. (**B**) Equal numbers of LCMV-specific CD8 T cells in LCMV-infected of *Trem1*+/+ or *Trem1*−/− mice at day 9 of infection, determined by staining with LCMV-gp33 loaded H-2D^b^ dextramer. Each dot represents the percentage of dextramer+ CD8+ T cells among all CD8+ T cells in the livers of individual mice. (**C**) Equal numbers of LCMV-responsive CD8 effector T cells in LCMV-infected of *Trem1*+/+ or *Trem1*−/− mice at day 9 of infection, determined by intracellular staining of CD8+ T cells for IFN-γ after stimulation with LCMV-gp33 peptide. Each dot represents the percentage of IFN-γ+ CD8+ T cells among all CD8+ T cells in the livers of individual mice. (**D**) Equal numbers of LCMV-responsive CD8 effector T cells in LCMV-infected of *Trem1*+/+ or *Trem1*−/− mice at day 9 of infection, determined by intracellular staining of CD8+ T cells for TNF-α after stimulation with LCMV-gp33 peptide. Each dot represents the percentage of TNF-α+ CD8+ T cells among all CD8+ T cells in the livers of individual mice. (**E**) Equal degranulation capacity of LCMV-responsive CD8 effector T cells in LCMV-infected of *Trem1*+/+ or *Trem1*−/− mice at day 9 of infection, determined by staining for CD107a after stimulation with LCMV-gp33 peptide. Each dot represents the percentage of CD107a+ CD8+ T cells among all CD8+ T cells in the livers of individual mice. (**F**) Similar LCMV-specific cytotoxic activity of CD8 effector T cells isolated from LCMV-infected *Trem1*+/+ (white bars) or *Trem1*−/− mice (black bars) at day 9 of infection, as determined by specific lysis (mean and SEM) of LCMV-infected Hepa1-6 hepatoma cells.

Since viral clearance is predominantly mediated by CD8+ T cells, we next analysed whether the accelerated clearance of LCMV in TREM1 deficient mice was associated with a more vigorous CD8 T cell response to LCMV (Fig. 3). Therefore, we first determined the recruitment of CD8+ T cells to infected livers in *Trem1*−/− and wild-type mice at day 9 of LCMV infection, when the difference in viral clearance was most prominent. There was no difference in the overall CD8 T cell numbers in the livers of *Trem1*−/− or wild-type mice (Fig. 3A; p = 0.4644). Moreover, there was no difference in the numbers of LCMV-specific CD8 T cells between the livers of wild-type or *Trem1*−/− mice (Fig. 3B; p = 0.8796), as determined by staining with LCMV-gp33 loaded H-2D^b^ dextramers by flow cytometry. We then analysed the antiviral effector functions of liver-infiltrating CD8+ T cells in response to stimulation with the immunodominant LCMV-gp33 peptide. To that end, we stained liver-infiltrating CD8+ T cells that were stimulated with the LCMV-gp33 peptide for intracellular IFN-γ (Fig. 3C) or TNF-α (Fig. 3D); there was no difference in the LCMV-gp33 induced IFN-γ response (p = 0.3070) or the TNF-α response (p = 0.3784) between C57BL/6 mice and *Trem1*−/− mice. Moreover, we assessed the ability of the CD8+ T cells to degranulate in response to stimulation with the LCMV-gp33 peptide by staining for CD107a; there was no difference in degranulation capacity between LCMV-specific CD8 T cells in the livers of wild-type or *Trem1*−/− mice (Fig. 3E; p = 0.7779). Furthermore, we assessed the cytotoxic activity of CD8 T cells isolated from infected livers of C57BL/6 mice or *Trem1*−/− mice in an *in vitro* cytotoxicity assay. We did not find a significant difference in the capacity to lyse target cells between the CD8 T cells derived from infected C57BL/6 mice or *Trem1*−/− mice (Fig. 3F). Thus, TREM1 deficiency was not associated with an altered CD8 T cell response to LCMV.

### Expression of TREM1 in LCMV-infected livers of wild-type mice is predominantly confined to neutrophils

**Figure 4:**
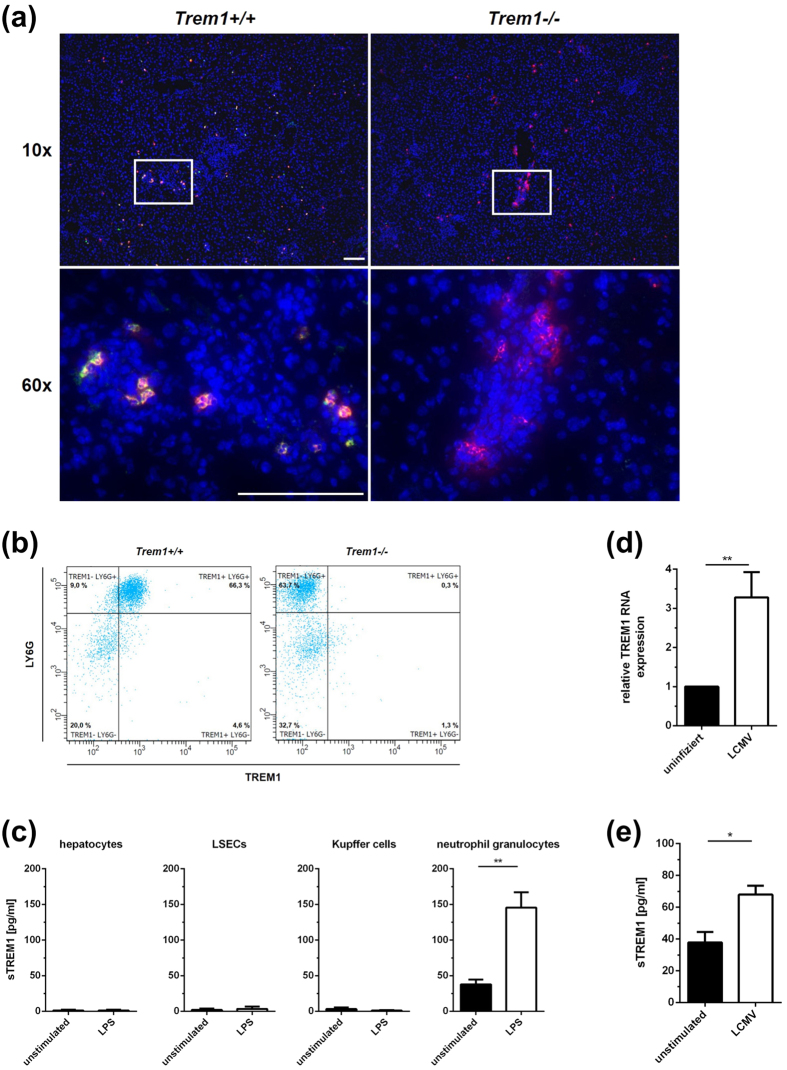
TREM1 expression in LCMV-infected livers of mice by neutrophils. (**A**) Frozen liver sections of C57BL/6 mice 9 days after infection with LCMV were stained for TREM1 (green) and Ly6G (red); nuclei were stained with Hoechst 33258 (blue). Co-expression of Ly6G (red) by TREM1-expressing cells (green) in livers of LCMV-infected mice confirmed that TREM1-expressing cells are neutrophils. Scale bar represents 100 μm. (**B**) Flow cytometric analysis of TREM1 and LY6G expression on neutrophil granulocytes isolated from *Trem1*+/+ und *Trem1*−/− C57BL/6 mice 9 days after infection with LCMV. (**C**) Concentrations of sTREM1 in culture supernatants of primary hepatocytes, liver sinusoidal endothelial cells (LSECs), Kupffer cells or neutrophils 24 hours after isolation from *Trem1*+/+ C57BL/6 mice and stimulation with LPS (10 ng/ml). Shown are mean values ± SEM. **p < 0.01. (**D**) Relative TREM1 RNA expression in neutrophils 6 hours after isolation from *Trem1*+/+ C57BL/6 mice and stimulation with LCMV WE (MOI 5). Shown are mean values ± SEM; **p < 0.01. (**E**) Release of sTREM1 by neutrophils into cell culture supernatant determined 24 hours after isolation from *Trem1*+/+ C57BL/6 mice and stimulation with LCMV WE (MOI 5). Shown are mean values ± SEM. **p < 0.01.

We next analysed histological liver sections by fluorescence microscopy to identify the TREM1-expressing cell types in the livers of LCMV-infected wild-type mice. As the TREM1-staining cells seemed to have polymorphic nuclei (Supplementary Fig. S1), we suspected neutrophils of being the major TREM1-expressing cell type. To validate this assumption, we performed co-staining for TREM1 and the neutrophil marker Ly6G (Fig. 4A), showing a high degree of co-expression. In contrast, co-staining of TREM1 with F4/80 (Supplementary Fig. S2), Ly6C (Supplementary Fig. S3) or CD8 (Supplementary Fig. S4), as markers for Kupffer cells, inflammatory monocytes or cytotoxic T cells, did not result in overlaying fluorescence. Thus, TREM1 seemed to be mainly expressed by neutrophils. To confirm TREM1 expression by neutrophils, we isolated Ly6G-expressing cells from the livers of LCMV-infected wild-type or *Trem1*−/− mice and analysed TREM1 expression by flow cytometry (Fig. 4B), showing that the majority of wild-type Ly6G^high^ neutrophils indeed expressed TREM1, whereas Ly6G^low^ monocytes did not express TREM1. Thus, TREM1 expression in the LCMV-infected livers was predominantly confined to neutrophils.

To learn whether neutrophils were also responsible for the shedding of sTREM1, we stimulated neutrophils *in vitro* for 24 hours with LPS, a known activator of neutrophils; as control, we used unstimulated neutrophils and various liver cell types that are alleged TREM1 expressors (Kupffer cells, liver sinusoidal endothelial cells or hepatocytes). None of the liver cells shed sTREM1 with or without LPS stimulation (Fig. 4C); in contrast, neutrophils released sTREM1, notably after LPS stimulation (Fig. 4C). We then tested whether stimulation with LCMV could likewise induce expression of TREM1 and shedding of sTREM1 by neutrophils. Indeed, we found that incubation of neutrophils with LCMV greatly up-regulated both TREM1 expression (Fig. 4D; p = 0.0078) and release of sTREM1 (Fig. 4E; P = 0.0211).

In an attempt to confirm that neutrophils were a major source of sTREM1 in LCMV-infected mice, we daily treated LCMV-infected C57BL/6 wild-type mice with a depleting antibody to Ly6G (1A8). As expected, this treatment resulted in significantly reduced numbers of neutrophils in the blood of treated mice; unexpectedly, however, this treatment did not induce effective depletion of neutrophils from the liver (Supplementary Fig. S5). Therefore, it was not possible to clarify without ambiguity whether neutrophils contribute to elevated sTREM1 levels *in vivo*. Nonetheless, our findings collectively suggested that, at least *in vitro*, neutrophils are activated by incubation with LCMV, to which they respond with up-regulated expression of TREM1 and shedding of sTREM1.

### TREM1 deficiency impairs secretion of CCL2 and TNF-α by neutrophils in response to LCMV

**Figure 5:**
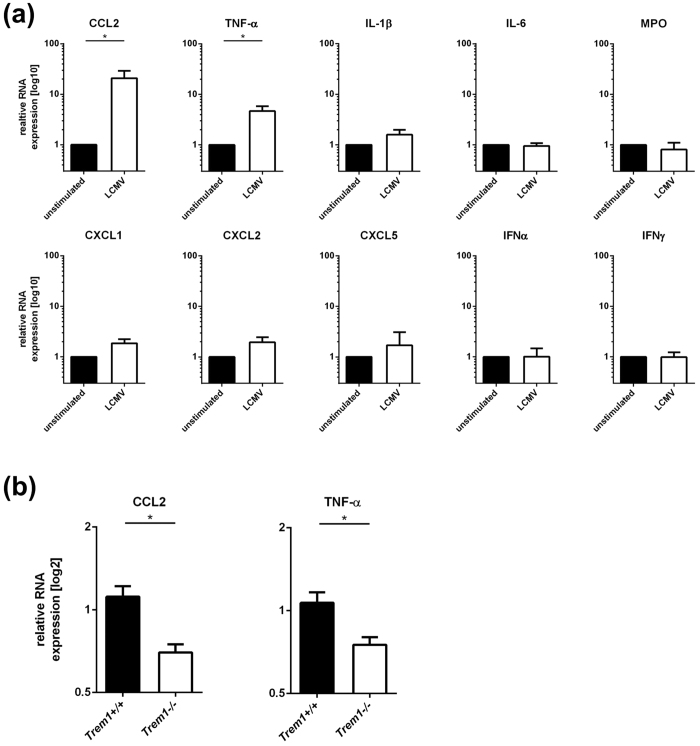
Impaired secretion of CCL2 and TNF-α by TREM1−/− neutrophils in response to LCMV. (**A**) Neutrophils were isolated from *Trem1*+/+ C57BL/6 mice, stimulated with LCMV WE (MOI 5) and, after 6 hours, the expression of CCL2, TNF-α, IL-1β, IL-6, MPO, CXCL1, CXCL2, CXCL5, IFN-α and IFN-γ relative to the HPRT house-keeper were determined by qRT-PCR. Shown are mean values ± SEM. *p < 0.05. (**B**) Relative CCL2 and TNF-α RNA expression in livers from *Trem1*+/+ and *Trem1*−/− C57BL/6 mice, sampled 4 days after infection with LCMV. Shown are mean values ± SEM. *p < 0.05.

We then addressed the question whether stimulation with LCMV could induce secretion of inflammatory mediators by neutrophils. To that end, we first analysed LCMV-stimulated wild-type neutrophils for the production of CCL2, TNF-α, IL-6, IL-1β, MPO, CXCL1, CXCL2, CXCL5, IFNα and IFNγ, all of which can be produced by neutrophils. As shown in Fig. 5A, the expression of CCL2 (p = 0.0053) and TNF-α (p = 0.0020), but not the other mediators was significantly up-regulated in neutrophils stimulated with LCMV. We then assessed whether TREM1 deficiency had influenced the LCMV-induced production of these mediators in LCMV hepatitis *in vivo* (Fig. 5B). Indeed, the production of the relevant mediators CCL2 and TNF-α was significantly reduced in *Trem1*−/− livers as compared to wild-type livers (p < 0.05), whereas the mediators that are not induced by LCMV (IL-6, IL-1β, MPO, CXCL1, CXCL2, CXCL5, IFNα and IFNγ) remained unchanged. Thus, TREM1 deficiency seemed to be associated with impaired secretion of CCL2 and TNF-α in LCMV hepatitis.

## Discussion

The goal of antiviral therapies is to achieve virus eradication from infected tissues with minimal pathology. To design better antiviral therapies, a more comprehensive understanding of the mechanisms underlying viral clearance and immunopathology is needed. Here, we investigated the role of TREM1 in viral hepatitis, as a modulating function of TREM1 in viral infections has been suspected^15^. Indeed we found that humans infected with HBV or HCV manifested elevated serum levels of sTREM1 (Fig. 1). Accordingly, mice infected with LCMV showed increased TREM1 expression in infected livers and increased shedding of sTREM1, notably in the phase of increased liver injury marked by elevated serum ALT (Fig. 1). As these findings seemed to confirm that TREM1 might play a role in viral hepatitis, we used *Trem1*−/− mice to study the possible function of TREM1 in viral liver infection. LCMV-infected *Trem1*−/− mice exhibited accelerated viral clearance from livers and reduced immunopathology, as compared to wild-type mice (Fig. 2). These effects were somewhat unexpected, because they indicate that viral clearance and immunopathology are at least to some extent independent outcomes of the anti-viral immune response. In fact, the liver pathology during viral hepatitis is caused by the immune response to virus^26^,^27^. Thus, one might assume that a more vigorous immune response that produces increased pathology will also produce accelerated viral clearance. However, we find here that the increased immunopathology in wild-type mice, as compared to *Trem1*−/− mice, was rather associated with protracted viral hepatitis and not accelerated clearance (Fig. 2). Although the protraction of viral hepatitis in wild-type mice was not very pronounced, this finding is all the more remarkable, because the increased expression of TREM1 during infection was associated with increased shedding of sTREM1, which can neutralize activating ligands and thus interfere with the pro-inflammatory function of the TREM1 receptor. Therefore, TREM1 signalling, even when restricted by sTREM1, seems to produce protracted infection accompanied with increased immunopathology. This finding is in line with a recent publication showing that TREM1 deficiency can attenuate infection-related disease severity^7^. Thus, TREM1 may be an attractive target to reduce the immunopathology of viral hepatitis without affecting, or even accelerating viral clearance.

To understand how TREM1 might cause increased immunopathology and yet protracted infection, we first analysed the anti-viral CD8 T cell response, because CD8 T cells are the major effector cells responsible for viral clearance from the liver^21^, but also for immunopathology^27^. As there were no apparent differences in the quantity and quality of the intra-hepatic anti-viral CD8 T cell response (Fig. 3), it is unlikely that TREM1 signaling had affected the adaptive immune response to LCMV. Instead, we found that TREM1 expression during viral hepatitis was essentially confined to liver-infiltrating neutrophils, which also seemed to be the major source of shed sTREM1 (Fig. 4 and Supplementary Figs S1–S4). It is therefore likely that neutrophils promoted the immunopathology of viral hepatitis by increasing liver damage in a non-specific manner. In fact, liver-infiltrating neutrophils have also been associated before with the immunopathology of liver infections^28^,^29^,^30^. As there were no apparent differences in neutrophil recruitment to infected livers between wild-type and *Trem1*−/− mice (Fig. 4), our findings suggest that the immunopathological activity of neutrophils depends on TREM1 signaling. Indeed, neutrophils responded to LCMV-stimulation mainly by producing CCL2 and TNF-α, and these were also the mediators that were significantly reduced in livers of infected *Trem1*−/− mice (Fig. 5). Thus, TREM1 seemed to aggravate the immunopathology of viral hepatitis, mainly by increasing the inflammatory activity of neutrophils.

Expression of TREM1 by various liver cell types, such as Kupffer cells^11^, sinusoidal endothelial cells^18^ and hepatic stellate cells^19^, has been reported. However, we could not detect any expression of TREM1 by these liver cells in immunohistochemical staining of infected livers (Fig. 4). Thus, the level of expression in these cells was probably too low to be detected by this method. Whether or not such low expression is of functional relevance is not entirely clear, but may be questioned. Indeed, we also did not detect any release of sTREM1 by various liver cells, whereas neutrophils that could well be stained for TREM1 in histology also readily released sTREM1. Therefore, we believe that neutrophils are much more relevant for the observed effects of TREM1-deficiency. Note, however, that it was not possible to confirm this notion through the depletion of neutrophils with a depleting antibody. Although antibody treatment resulted in a strong reduction of neutrophils in the blood, which is in accordance with published findings by others^31^,^32^, there was no significant reduction of neutrophils in the inflamed liver (Supplementary Fig. S5). Therefore, we cannot fully exclude that other cell types contribute to the observed TREM1-mediated pathology and the release of sTREM1; however, we believe that neutrophils are of major relevance for these effects.

Taken together, our findings indicate that TREM1 influences the course of and the immunopathology associated with viral hepatitis, mainly through the activity of neutrophils. Therefore, interference with the functions of TREM1 or neutrophils may be a therapeutic target enabling the treatment of viral infection-associated immunopathologies without affecting viral clearance.

## Methods

### Patients

Blood from patients with HBV or HCV infections was made available by the Institute of Medical Microbiology, Virology and Hygiene of the University Medical Centre Hamburg-Eppendorf. Patients were diagnosed according to the EASL (European Association for the Study of the Liver) practice guidelines for the management of HBV or HCV infections^33^,^34^. In summary, blood plasma from 34 HBV patients (median age: 42 years; range: 19–59 years; 71% male), 29 HCV patients (median age: 55 years; range: 39–77 years; 78% male) and 17 healthy controls (median age: 31 years; range: 27–45 years; 64% male) was subject to analysis of sTREM1. Median viremia in HBV and HCV patients was 1250 IU/ml and 700.000 IU/ml, respectively. Patients under treatment with pegylated interferon-α, or co-infection with HIV or HDV were excluded from this study.

### Mice

The TREM1 deficient mouse strain used for this research project (Trem1^tm1(KOMP)Vlcg^) was generated by the trans-NIH Knock-Out Mouse Project (KOMP) and obtained from the KOMP Repository (www.komp.org). NIH grants to Velocigene at Regeneron Inc (U01HG004085) and the CSD Consortium (U01HG004080) funded the generation of gene-targeted ES cells for 8500 genes in the KOMP Program and archived and distributed by the KOMP Repository at UC Davis and CHORI (U42RR024244). The mice were bred and kept in the animal facility of the University Medical Centre Hamburg-Eppendorf under specific pathogen-free conditions. *Trem1*+/+ control mice were TREM1-proficient littermates or C56BL/6 mice after backcross to this background for more than 10 generations in our facility. Mice were 8–15 weeks of age at the start of experiments. Animal experiments were conducted in accordance with institutional guidelines and approved by the review board of the State of Hamburg, Germany. For the quantification of liver injury, alanine aminotransferase (ALT) concentrations in mouse plasma were measured at the Institute of Clinical Chemistry and Laboratory Medicine of the University Medical Center Hamburg-Eppendorf, Hamburg, Germany.

### Virus

LCMV WE was originally provided by Dr. R. M. Zinkernagel (Swiss Institute of Technology, Zürich, Switzerland) and propagated on L929 mouse fibroblasts. Mice were infected as described^24^. As indicated, neutrophil depletion was performed by intraperitoneal application of 500 mg of 1A8 antibody (BioXcell) every second day starting 1 day prior LCMV inoculation. LCMV focus-forming units (FFU) were determined as described^35^, using the EnVision System^TM^ (DAKO) according to the manufacturer’s instructions.

### RNA isolation and gene expression analysis

Total RNA from mouse livers or neutrophils was extracted using TRIzol reagent (Life Technologies) or NucleoSpin TriPrep kit (Macherey-Nagel) according to the manufacturer’s instructions. Total RNA (1 μg) was used for cDNA preparation with the First Strand cDNA Synthesis Kit for RT-PCR (AMV) or High-Capacity cDNA Reverse Transcription Kit (Life Technologies) according to the manufacturer’s instructions. Real-time RT-PCR was performed on a StepOnePlus real-time PCR (polymerase chain reaction) system (Life Technologies) and expression was calculated by the ΔΔCt method^36^. Gene expression was normalized to the peptidylprolyl isomerase A (*PPIA*) housekeeper mRNA. TaqMan assay-on-Demand primer/probe sets (Life Technologies) were used for the detection of gene expression for TREM1: Mm01278455_m1, DAP12: Mm00449152_m1, PPIA: Mm02342429_g1, CCL2: Mm00441242_m1, TNF-α: Mm00443258_m1, IL-1β: Mm00434228_m1, IL-6: Mm00446190_m1, MPO: Mm01298424_m1, CXCL1: Mm04207460_m1, CXCL2: Mm00436450_m1, CXCL5: Mm00436451_g1, IFN-α: Mm03030145_gH and IFN-γ: Mm01168134_m1. LCMV Z RNA expression was quantified by using the LightCycler FastStart DNA Master SYBR Green I hot start reaction mix (Roche) on a LightCycler© 1.5 system (Roche) with custom-made primer pairs (LCMV_Z_fwd: 5′-CAGACACCACCTATCTTGG-3′ and LCMV_Z_rev: 3′ ACCTTCAG TTTGGTTGGC-5′).

### Cell isolation and cultivation

Mononuclear cells were obtained from C57BL/6 mice after perfusion of livers with 2 ml Ca^2+^-free PBS (2.7 mM KCl, 1.5 mM KH_2_PO_4_, 6.5 mM Na_2_HPO_4_ and 137 mM NaCl) and mechanical dissection of liver tissue followed by density gradient centrifugation (530 g; 20 min; 20 °C) of liver homogenates in 5 ml 40% Percoll (GE Healthcare), covered with 3 ml 70% Percoll. Red blood cells were lysed by hypertonic cell lysis in ACK-buffer (100 mM EDTA, 10 mM KHCO_3_ and 150 mM NH_4_Cl). For analysis of CD8+ T cell effector functions, mononuclear cells were cultured in 96-well microtiter plates (Sarstedt) in Iscove’s Modified Dulbecco’s Medium (IMDM) containing 25 mM HEPES, 10% FCS, 5% L-glutamine and 1% penicillin/streptomycin. For the cytotoxicity assay, CD8 T cells were enriched from liver and spleen homogenates of infected mice by magnetic separation according to the manufacturer (Miltenyi Biotech). The cytotoxicity assay was then performed as described^37^, with the modification that Hepa1-6 mouse hepatoma cells, which had been infected one day before with LCMV (0.1 MOI), were used as target cells. Specific cytotoxicity was calculated using the following formula: % specific lysis = (experimental–spontaneous release) × 100/(maximum–spontaneous release).

Primary hepatocytes were isolated from mouse livers as described^38^. Briefly, livers were perfused with 0.15 PZU/ml collagenase (Serva) in PBS containing 68 mM NaCl, 6.7 mM KCl, 100 mM HEPES and 4.8 mM CaCl_2_ × 2H_2_O followed by centrifugation of liver homogenates for 5 min at 30 g and 4 °C and subsequent lysis of red blood cells. Primary hepatocytes were cultured in collagen pre-treated 6-well microtiter plates in William’s E Medium (Life Technologies) supplemented with 10% FCS, 20 mM HEPES, 0.02% insulin, 5% L-glutamine and 1% penicillin/streptomycin.

Kupffer cells and LSECs were isolated from mouse livers as described^39^ by perfusion of livers with 0.5% collagenase in 5 ml Gey’s balanced salt solution (GBSS) (5.5 mM (D+) glucose, 1.6 mM CaCl_2_ × 2H_2_O, 10 mM HEPES, 5 mM KCl, 0.2 mM KH_2_PO_4_, 0.9 mM MgCl_2_ × 6H_2_O, 0.3 mM MgSO_4_ × 7H_2_O, 1.7 mM Na_2_HPO_4_, 137 mM NaCl and 2.7 mM NaHCO_3_) followed by mechanical dissection of liver tissue, ACK-lysis of red blood cells and density gradient centrifugation of liver cells in 2 ml OptiPrep^TM^ (Sigma-Aldrich) (430 g; 40 min; 4 °C), covered with 1 ml PBS. Kupffer cells and LSECs were separated from other cells by positive magnetic cell isolation (MACS) using Kupffer-cell-specific anti-F4/80 (CI:A3-1; Biolegend) and LSEC-specific anti-CD146 (ME-9F1; Biolegend) monoclonal antibodies and antibody-specific magnetically-labelled microbeads (Miltenyi Biotec).

Neutrophil granulocytes were isolated from mouse liver and spleen homogenates by perfusion of livers with PBS and mechanical dissection of organs followed by centrifugation of organ homogenates (300 g; 10 min; 4 °C), ACK-lysis of red blood cells and negative magnetic cell isolation using the Neutrophil Isolation Kit (Miltenyi Biotec) according to the manufacturer’s instructions.

Kupffer cells (1*10^5 ^cells/well), LSECs (1*10^5 ^cells/well) and neutrophil granulocytes (2*10^5 ^cells/well) were cultured in 96-well microtiter plates in IMDM supplemented with 25 mM HEPES, 10% FCS, 5% L-glutamine and 1% penicillin/streptomycin. For stimulation experiments, cell culture medium was substituted with 10 ng/ml LPS (L2880; Sigma-Aldrich), 5 MOI (multiplicity of infection) LCMV WE.

### Flow Cytometry

Live/dead cell staining was performed with Pacific OrangeTM, succinimidyl ester, triethylammonium salt (Life Technologies) in PBS. Immunofluorescent surface staining of CD8+ T cells was performed in PBS with 2% BSA and fluorochrome-conjugated antibodies specific for CD8, CD107a, TNF-α or IFN-γ (BioLegend), or with fluorochrome-conjugated dextramers specific for the immunodominant H-2D^b^ restricted gp33-41 (KAVYNFATC) LCMV peptide (Immudex), as described^24^. Stained cells were fixed overnight in 1% PFA in PBS. For staining of CD107a, intracellular IFN-γ or intracellular TNF-α, cells were stimulated for 4 hours with the immundominant gp33-41 (KAVYNFATC) LCMV peptide (3 μg/ml) in the presence of 1 μl/ml Golgi-Plug (BD Bioscience) or 0.65 μl/ml Golgi Stop (CD107a) (BD Bioscience) in Panserin 401 medium (PAN Biotec), supplemented with 1% penicillin/streptomycin and 0.5 × 104 M β-mercaptoethanol. For intracellular staining of IFN-γ and TNF-α, cells were permeabilized in buffer containing 2% BSA and 0.5% saponin and stained with antibodies against IFN-γ and TNF-α. For immunofluorescent surface staining of TREM1 or LY6G on neutrophils, MACS-isolated cells were incubated with fluorchrome-labelled anti-TREM1 polyclonal antibody AF1187 (R&D) or anti-LY6G PE-conjugated monoclonal primary antibody 1A8 (Biolegend) in PBS for 10 min at 4 °C. Flow cytometry was performed with an LSR II cytometer (BD Bioscience) and data was analysed with FACSDiva 6.0 Software (BD Bioscience).

### Liver histology and immunohistochemistry

At the indicated time points, mice were sacrificed and the livers were fixed in 4% neutral-buffered formaldehyde, embedded in paraffin, sectioned at 3 μm, and stained with hematoxylin and eosin (H&E). Necroinflammatory activity in paraffin-embedded, H&E-stained liver sections was scored in a blinded fashion by a pathologist (T. K.) using the modified histological activity index (mHAI). The mHAI is a standardized score for grading the severity of interface hepatitis, confluent necrosis, single cell necrosis and portal inflammation in viral hepatitis^40^.

Alternatively, mouse livers were embedded in Tissue-Tek (Sakura), snap-frozen and stored at 80 °C. Tissue sections (8 μm) were fixed in acetone for 10 min and blocked with 1% BSA, 5% normal rat serum and anti-CD16/anti-CD32 antibody (eBioscience; 1:50) in PBS. Anti-TREM1 polyclonal primary antibody AF1187 (R&D; 1:300), anti-LY6G PE-conjugated monoclonal primary antibody 1A8 (Biolegend; 1:300) or secondary Alexa-488-conjugated anti-LCMV NP-specific monoclonal antibody VL4 (Bio X cell; 1:100) were applied for 1 h at room temperature. For specific staining of TREM1, the slides were incubated for 1 h with a goat-specific Alexa 488-conjugated secondary antibody (Invitrogen; 1:1300). All slides were counterstained for 1 min with Hoechst 33258, pentahydrate (bis-Benzimide) (Sigma-Aldrich;) nuclear dye 1:10.000 in PBS. Analysis of immunofluorescence was performed with a BZ-9000 microscope system (Keyence) using the software BZ-II Analyzer (Keyence).

### ELISA

sTREM1 concentrations in plasma from HBV/HCV patients, urine and plasma from mice as well as primary cell culture supernatants were measured with the TREM1 DuoSet (R&D) according to the manufacturer’s instructions.

### Statistics

Data are presented as mean ± SEM (standard error of mean) or median values. Statistical significance of differences between two groups was determined by Student’s t-test. Differences of numerical variables between more than two groups were assessed by one-way ANOVA and Dunn’s multiple comparisons test. A p-value of p ≤ 0.05 was considered to be statistically significant.

## Additional Information

**How to cite this article**: Kozik, J.-H. *et al*. Attenuated viral hepatitis in *Trem1*−/− mice is associated with reduced inflammatory activity of neutrophils. *Sci. Rep.* **6**, 28556; doi: 10.1038/srep28556 (2016).

## Supplementary Material

Supplementary Information

## Figures and Tables

**Table 1:** Characteristics of patients with viral liver disease and healthy controls.

	n	age [years] median (min/max)	sex [%] (male/female)	viral load [IU/ml] mean (min/max)
Healthy	17	31 (26**/**45)	65**/**35	− −/−
HBV	34	42 (34**/**59)	71**/**29	9,67*10^7^ (30**/**8*10^8^)
HCV	29	52 (46**/**77)	78**/**22	1,31*10^7^ (20000**/**8*10^7^)

IU = international units; min = minimum; max = maximum.
