# Supplementary material for: Attenuated viral hepatitis in Trem1−/− mice is associated with reduced inflammatory activity of neutrophils
Source: Sci Rep. 2016 Jun 22;6:28556. doi: 10.1038/srep28556 (PMC4916511; doi:10.1038/srep28556)

Attenuated viral hepatitis in *Trem1-/-* mice is associated with reduced inflammatory activity of neutrophils

Jan-Hendrik Kozik[1], Tanja Trautmann[1], Antonella Carambia[1], Max Preti[1], Marc Lütgehetmann[2], Till Krech[3], Christiane Wiegard[1], Joerg Heeren[4], Johannes Herkel[1,]*

[1]University Medical Centre Hamburg-Eppendorf, Department of Medicine I, 20246 Hamburg, Germany
[2]University Medical Centre Hamburg-Eppendorf, Institute of Medical Microbiology, Virology and Hygiene, 20246 Hamburg, Germany
[3]University Medical Centre Hamburg-Eppendorf, Institute of Pathology, 20246 Hamburg, Germany
[3]University Medical Centre Hamburg-Eppendorf, Institute of Biochemistry and Molecular Cell Biology, 20246 Hamburg, Germany

*Correspondence to jherkel@uke.de

Supplementary Information

Supplementary Figure S1

Frozen liver sections of C57BL/6 mice 9 days after infection with LCMV were stained for TREM1 (green) and nuclei were stained with Hoechst 33258 (blue), revealing that TREM1-expressing cells have polymoprphic nuclei. The scale bar indicates a distance of 100 μm.

Trem1+/+

60x

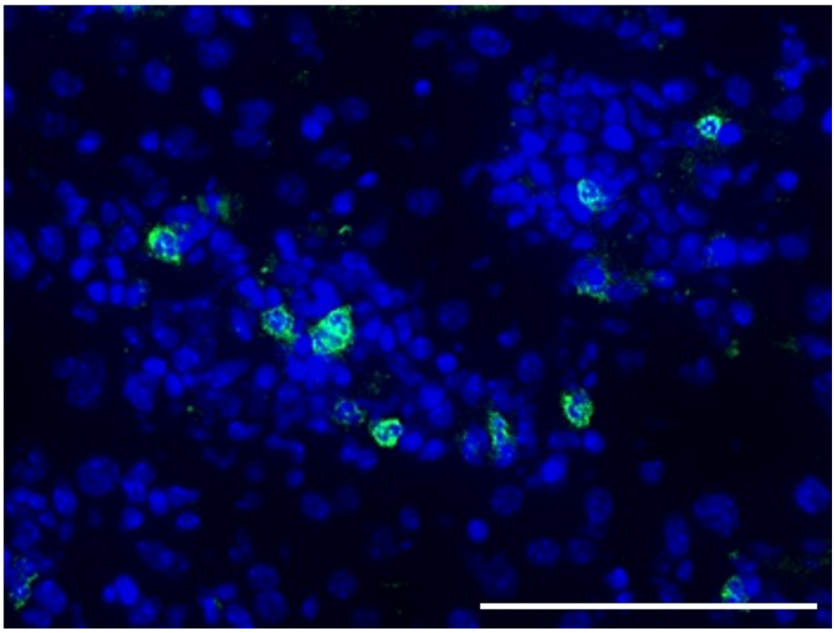

Supplementary Figure S2

Frozen liver sections of C57BL/6 mice 9 days after infection with LCMV were stained for TREM1 (green) and F4/80 (red); nuclei were stained with Hoechst 33258 (blue). The scale bar indicates a distance of 100 μm.

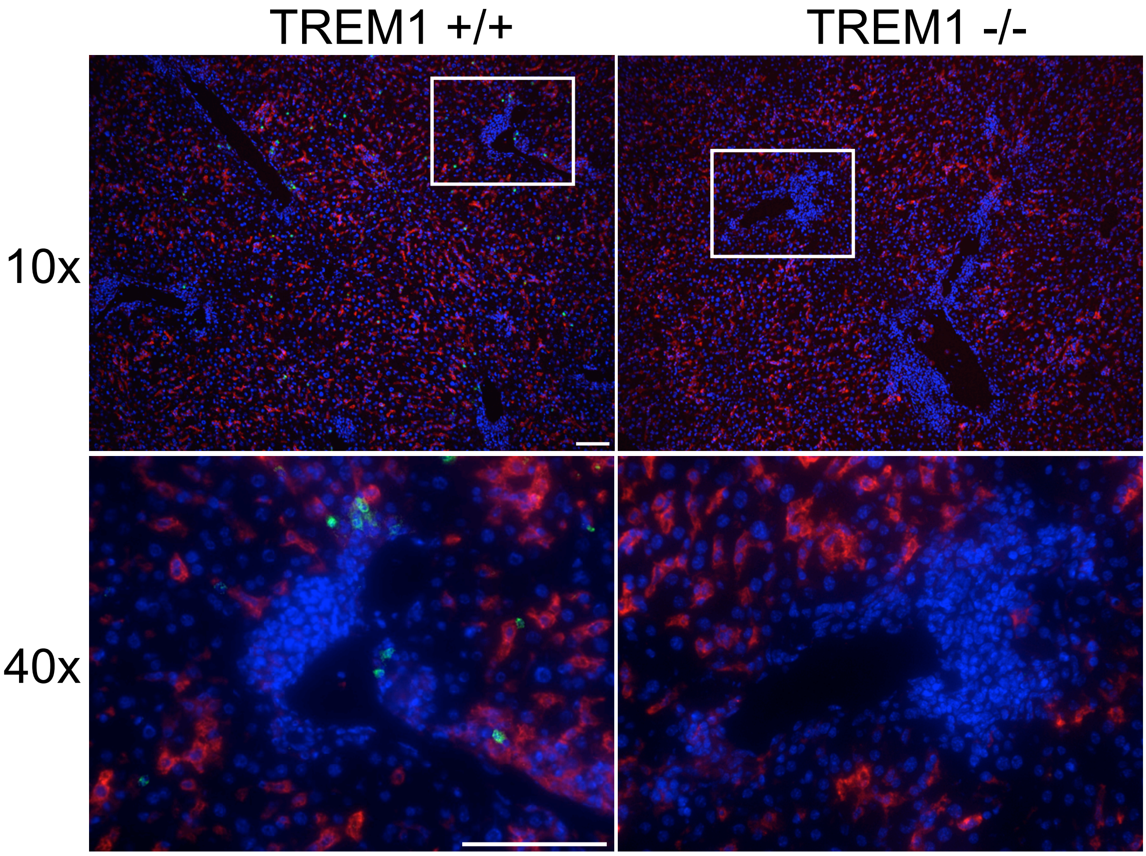

Supplementary Figure S3

Frozen liver sections of C57BL/6 mice 9 days after infection with LCMV were stained for TREM1 (green) and Ly6C (red); nuclei were stained with Hoechst 33258 (blue). The scale bar indicates a distance of 100 μm.

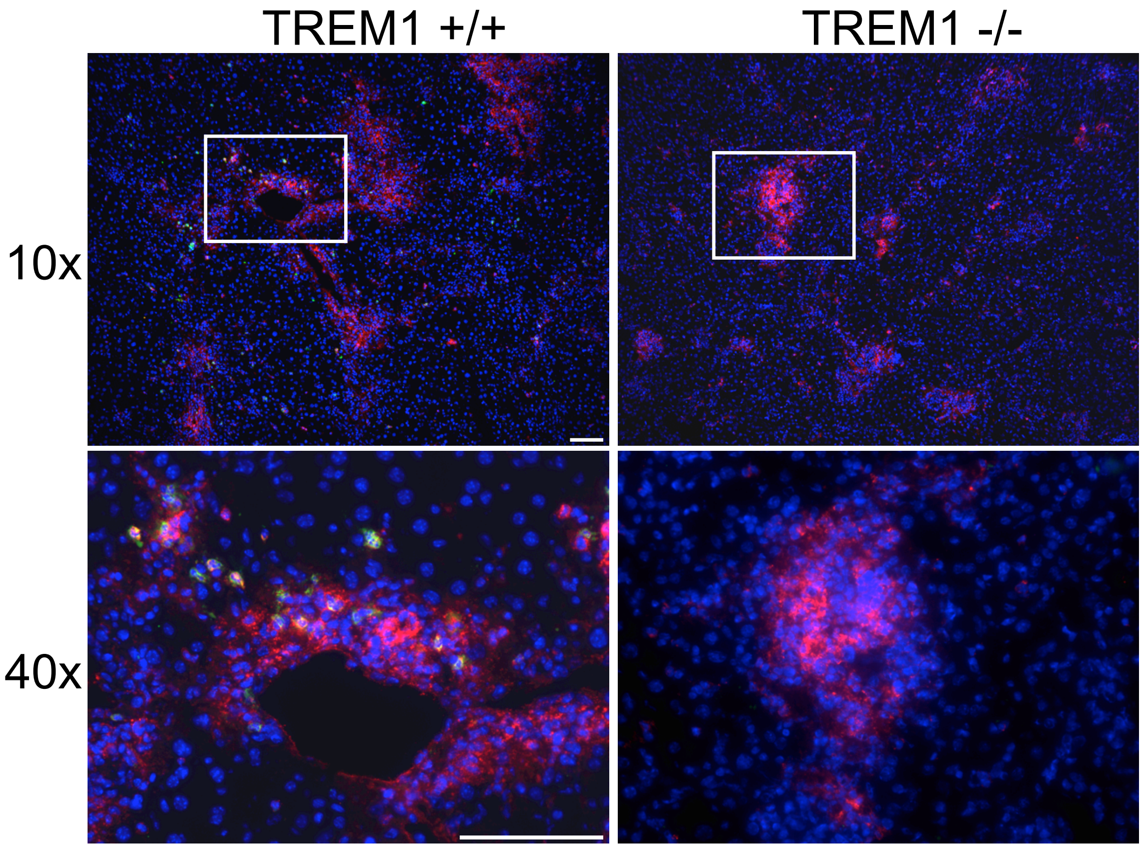

Supplementary Figure S4

Frozen liver sections of C57BL/6 mice 9 days after infection with LCMV were stained for TREM1 (green), CD8 (red) and CD4 (cyan); nuclei were stained with Hoechst 33258 (blue). The scale bar indicates a distance of 100 μm.

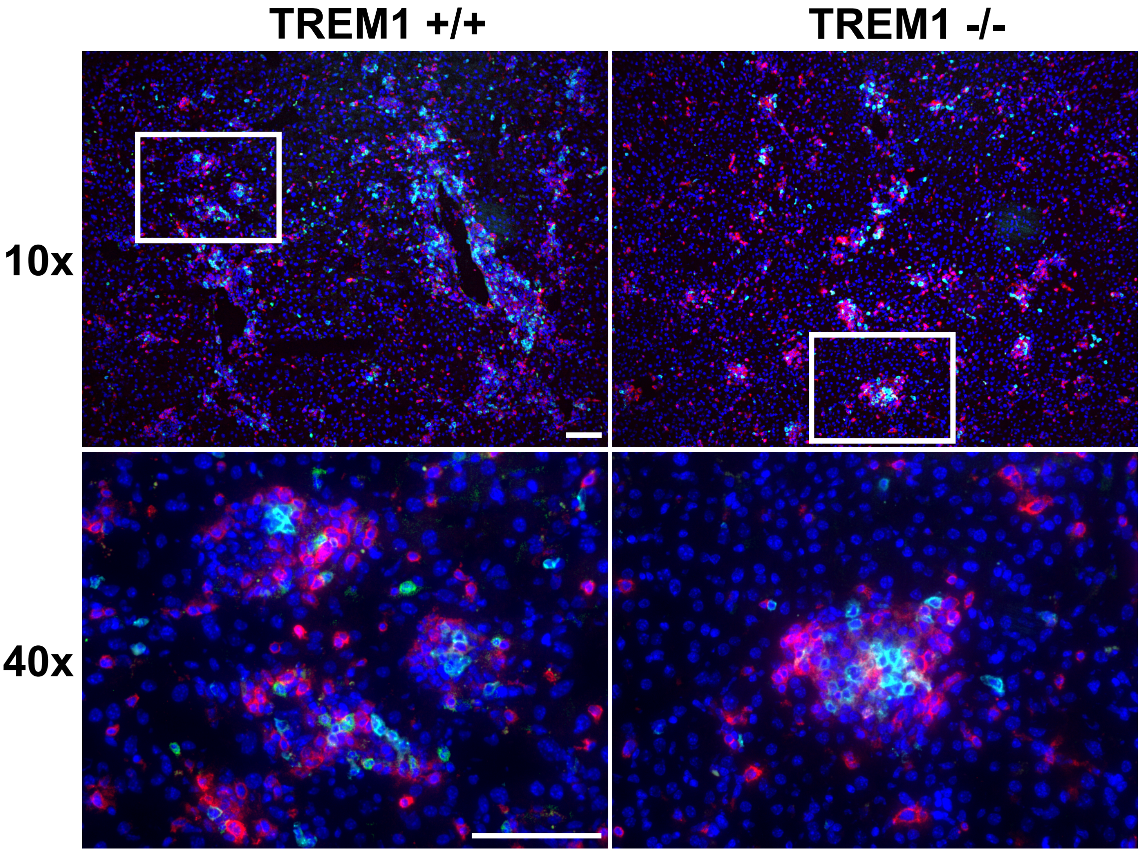

Supplementary Figure S5

LCMV-infected C57BL/6 mice were repeatedly treated with neutrophil-depleting antibody (αLy6G) or isotype-matched control antiobody. Frozen liver sections of these mice 9 days after infection with LCMV were stained for TREM1 (green) and Ly6G (red); nuclei were stained with Hoechst 33258 (blue). The scale bar indicates a distance of 50 μm.

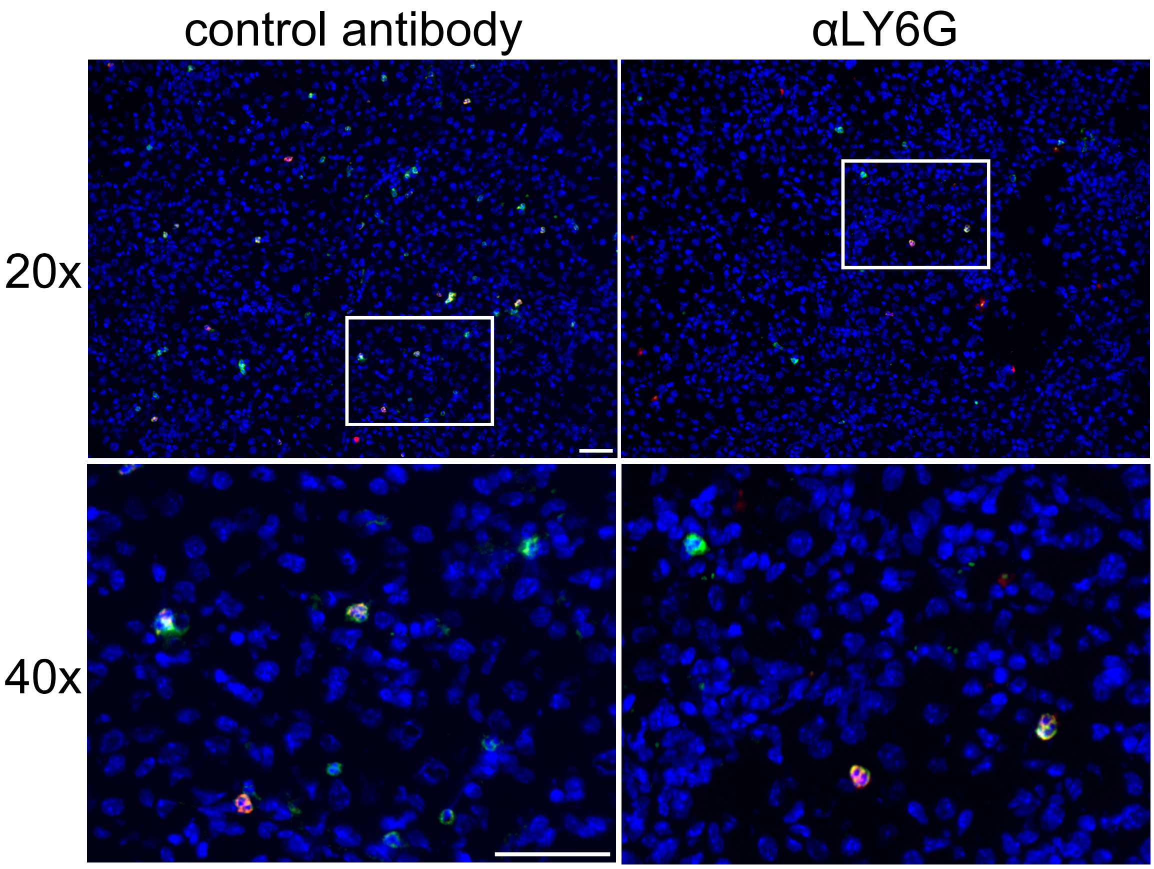

Supplement: Supplementary Information [file srep28556-s1.pdf]
